# Cytochrome *bd*-I in *Escherichia coli* is less sensitive than cytochromes *bd*-II or *bo*′' to inhibition by the carbon monoxide-releasing molecule, CORM-3^☆^^☆☆^: N-acetylcysteine reduces CO-RM uptake and inhibition of respiration

**DOI:** 10.1016/j.bbapap.2013.04.019

**Published:** 2013-09

**Authors:** Helen E. Jesse, Tacita L. Nye, Samantha McLean, Jeffrey Green, Brian E. Mann, Robert K. Poole

**Affiliations:** a Department of Molecular Biology and Biotechnology, The University of Sheffield, Sheffield, S10 2TN, UK; b Department of Chemistry, The University of Sheffield, Sheffield, S10 2TN, UK

**Keywords:** CO-RM, carbon monoxide-releasing molecule, CORM-2, ([Ru(CO)_3_Cl_2_]_2_), CORM-3, Ru(CO)_3_Cl(glycinate), DCFH-DA, (2′7′-diacetate), EDTA, ethylene diamine tetraacetic acid, EGTA, ethylene glycol tetraacetic acid, HO-1, haem oxygenase-1, iCORM-3, inactive CORM-3, ICP-MS, inductively coupled plasma mass spectrometry, *K*_d_, dissociation constant, K*_L_a*, gas transfer (gas to liquid) coefficient, *K*_m_, Michaelis constant, the concentration of substrate that gives half-maximal velocity, LB, Luria Bertani broth, miCORM-3, myoglobin-inactivated CORM-3, NAC, N-acetylcysteine, PBS, phosphate-buffered saline, ROS, reactive oxygen species, SOD, superoxide dismutase, *V*_max_, maximal rate, CORM-3, CORM-2, Cytochrome, *Escherichia coli*, Respiratory oxidase, N-acetylcysteine

## Abstract

Background: CO-releasing molecules (CO-RMs) are potential therapeutic agents, able to deliver CO – a critical gasotransmitter – in biological environments. CO-RMs are also effective antimicrobial agents; although the mechanisms of action are poorly defined, haem-containing terminal oxidases are primary targets. Nevertheless, it is clear from several studies that the effects of CO-RMs on biological systems are frequently not adequately explained by the release of CO: CO-RMs are generally more potent inhibitors than is CO gas and other effects of the molecules are evident. Methods: Because sensitivity to CO-RMs cannot be predicted by sensitivity to CO gas, we assess the differential susceptibilities of strains, each expressing only one of the three terminal oxidases of *E. coli —* cytochrome *bd*-I, cytochrome *bd*-II and cytochrome *bo′*, to inhibition by CORM-3. We present the first sensitive measurement of the oxygen affinity of cytochrome *bd*-II (*K*_m_ 0.24 μM) employing globin deoxygenation. Finally, we investigate the way(s) in which thiol compounds abolish the inhibitory effects of CORM-2 and CORM-3 on respiration, growth and viability, a phenomenon that is well documented, but poorly understood. Results: We show that a strain expressing cytochrome *bd*-I as the sole oxidase is least susceptible to inhibition by CORM-3 in its growth and respiration of both intact cells and membranes. Growth studies show that cytochrome *bd*-II has similar CORM-3 sensitivity to cytochrome *bo′*. Cytochromes *bo′* and *bd*-II also have considerably lower affinities for oxygen than *bd*-I. We show that the ability of N-acetylcysteine to abrogate the toxic effects of CO-RMs is not attributable to its antioxidant effects, or prevention of CO targeting to the oxidases, but may be largely due to the inhibition of CO-RM uptake by bacterial cells. Conclusions: A strain expressing cytochrome *bd*-I as the sole terminal oxidase is least susceptible to inhibition by CORM-3. N-acetylcysteine is a potent inhibitor of CO-RM uptake by *E. coli*. General significance: Rational design and exploitation of CO-RMs require a fundamental understanding of their activity. CO and CO-RMs have multifaceted effects on mammalian and microbial cells; here we show that the quinol oxidases of *E. coli* are differentially sensitive to CORM-3. This article is part of a Special Issue entitled: Oxygen Binding and Sensing Proteins.

## Highlights

• Cytochrome *bd*-I is a CORM- insensitive heme-protein in *E. coli*.
• The oxygen affinity of the ‘third oxidase’, cytochrome *bd*-II is low (K_m_ 0.24 μM).
• Non-thiol antioxidants do not prevent CO-RM-mediated inhibition of respiration.
• N-acetylcysteine reduces the uptake of CORM-2 and CORM-3 by *E. coli*.

## 1 Introduction

Carbon monoxide (CO) inhibits respiration by binding to haems, particularly the terminal oxidases and globins of aerobic respiration [1]. However, since 1991 [2], CO has come to be recognised as an important endogenous gas with beneficial roles at low concentrations [3]. CO is produced endogenously by haem oxygenases, functions as a neural messenger [4] and also has vasodilatory [5], anti-inflammatory and anti-apoptotic properties [6].

The discovery of the beneficial effects of low concentrations of CO has prompted research into the use of CO as a human therapeutic agent [7]. Due to the obvious dangers of CO inhalation therapy, CO carriers, analogous to NO-releasing compounds, were developed specifically for medical applications; the first such report was a patent submission in 2001 [8]. These carbon monoxide-releasing molecules, or CO-RMs, have diverse biological effects, similar in many respects, but not identical, to those of CO [9], including vasodilation [10–12], reducing inflammation [13,14] and cardiac graft rejection [15,16].

However, it is important to note that numerous studies show clearly that the biological effects of CO-RMs cannot be wholly explained by the liberation of CO. That is, the effects of CO-RMs are not precisely replicated by additions of CO gas solutions. For example, Nobre [17] showed that both CORM-2 and CORM-3 were more potent antibacterial agents than CO gas. Additionally, CORM-3 is inhibitory to bacterial growth at micromolar concentrations and at atmospheric oxygen levels [18], whereas it is generally understood that CO must be present in significant excess over oxygen [1] (typically in a ratio of 19:1) [19] to be inhibitory. Furthermore, even saturated solutions of CO gas (approx. 1 mM) are known to allow bacterial growth [20] whereas only 100 μM CORM-3 or less is highly toxic to aerobic *E. coli* cultures [18]. CORM-3 also inhibits respiration of cells, after transiently accelerating oxygen uptake by an uncoupler-independent mechanism, and promotes cation transport across spheroplast membranes, yet these effects are not mimicked by CO gas [21].

Previous work has shown that a range of CO-RMs are bactericidal against *Staphylococcus aureus* and *E. coli* [17] and that CORM-3 increases survival in mice infected with *Pseudomonas aeruginosa* [22]. We previously identified the aerobic respiratory chain as a direct bacterial target in *E. coli* by demonstrating that CO from CORM-3 is delivered directly to intracellular cytochromes *bd* and *bo′* of the aerobic respiratory chains [18]. Further evidence that the inhibition of respiration is due mainly to binding of CO from CO-RMs to oxidases comes from the reversal of CORM-3-mediated respiratory and growth inhibition by photolysis of the haem–CO bond [21].

However, unexpected, non-haem protein targets exist, such as, in bacteria, diverse transport processes [21] and the metabolism, homeostasis, and transport of metals [18]. In higher cells, CO activates large-conductance Ca^2 +^- and voltage-gated K^+^ (Slo1 BK) channels, which are involved in oxygen sensing, vasodilation, and the activation of signalling from neurones [23]. The interaction of CO with such channels involves sulfur from cysteine and nitrogen from histidine residues within the cytoplasmic domain of the channel. CO is thought to act as a partial agonist for the divalent cation sensor in the Slo1 BK channel [24]. CO is also known to bind to iron in Fe, Fe–Fe and Fe–Ni hydrogenases [25], to the Fe–Fe hydrogenases of *Chlamydomonas* [26] and to binuclear copper sites as in tyrosinase [27] and haemocyanins [28,29].

It has been noted in several studies that the thiol compound N-acetylcysteine (NAC) abolishes the biological activities of metal-containing CO-RMs, preventing induction of HO-1 expression and haem oxygenase activity in murine macrophages by CORM-3 [13], and relieving inhibitory effects of CORM-2 on the mitochondrial respiratory chain and NAD(P)H oxidase [30]. More recently, several thiol compounds have been found to prevent the effects of CORM-3 on the growth and oxygen consumption of *P. aeruginosa* [22]. Several hypotheses have been proposed to explain this. Tavares et al. [31] have suggested that such compounds nullify the activity of CO-RMs by virtue of their antioxidant properties, as high concentrations of CORM-2 cause an increase in reactive oxygen species (ROS), which is diminished by exogenous glutathione. Desmard et al. [22] also investigated this possibility but found that 100 μM CORM-2, CORM-3, CORM-371 or CORM-A1 did not cause any ROS production in *P. aeruginosa* within 1 h of treatment as measured by DCFH-DA (2′7′-dichloroflurescin diacetate).

Terminal oxidases may be prime determinants of sensitivity to CO-RMs in their antimicrobial activities. The three terminal oxidases of *E. coli* have distinctive properties. It is tacitly assumed that these differences in affinity for oxygen [32], contributions to proton translocation [33] and patterns of expression as a result of transcriptional regulation [34,35] are reflected in their physiological roles and fitness for particular environmental niches. Here we assess the differential susceptibilities of bacteria possessing each of the three terminal oxidases of *E. coli –* cytochromes *bd*-I, *bd*-II and *bo′* – to CORM-3. We report growth studies and measurements of respiration in the presence of CORM-3. We also test the hypothesis that N-acetylcysteine may lower CO-RM uptake, and suggest that this probably explains, in part, the ability of the thiol to interfere with the toxic effects of both CORM-2 and CORM-3.

## 2 Materials and methods

### 2.1 *E. coli* strains and growth conditions

All *E. coli* strains used were K-12 derivatives; MG1655 (RKP5416) was the wild type [36] from which the respiratory mutants, TBE023, referred to throughout this paper as the parent strain (MG1655 Δ *nuoB*::kan), TBE025 referred to as Cyo^+^ (MG1655 Δ*cydB nuoB appB*::kan), TBE026 referred to as App^+^ (MG1655 Δ*cydB nuoB cyoB*::kan) and TBE037 referred to as Cyd^+^ (MG1655 Δ*appB nuoB cyoB*::kan) were derived (mutants kindly given by Alex Ter Beek and Joost Teixeira de Mattos, University of Amsterdam). These strains carry the same mutant alleles as described by Bekker et al. [37]. Unless stated otherwise, *E. coli* cultures were grown aerobically in defined minimal medium with glycerol (54 mM) as a carbon source [18]. For growth studies, cultures were grown with or without 12.5 μM CORM-3 (added at an optical density of 30 Klett units) in 30 ml medium in 250 ml conical flasks fitted with side arms for measurements of optical density with a Klett meter with a no. 66 (red) filter (Klett Manufacturing Co., New York, N.Y.). Cultures were incubated at 37 °C and shaken at 200 rpm.

### 2.2 Preparation of *E. coli* membrane particles

*E. coli* cells were grown in 1 l LB broth in 2 l baffled flasks at 37 °C with shaking at 250 rpm until mid-exponential phase. Membranes were prepared as described previously [38]. Protein concentrations were measured using a modified Lowry procedure [39].

### 2.3 CO-RM and control treatments

**Fig. 1:**
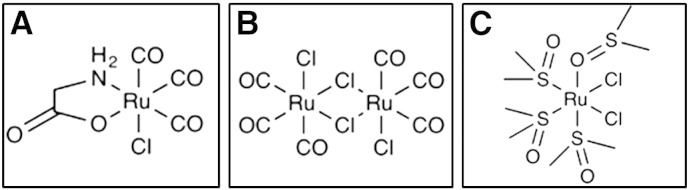
The structure of the CO-RM compounds used in this work. (A) CORM-3, (B) CORM-2 and (C) RuCl_2_(DMSO)_4_, the compound used as a control for experiments with CORM-2.

The CO-RMs used in this study were tricarbonyldichlororuthenium(II) dimer ([Ru(CO)_3_Cl_2_]_2_), referred to as CORM-2 [40] and Ru(CO)_3_Cl(glycinate), referred to as CORM-3 [15]; the structures of these compounds are shown in Fig. 1A and B. CORM-2 was purchased from Sigma Aldrich and prepared as a 10 mM stock solution in DMSO. CORM-3 was synthesised as previously described [41]. Stock solutions (10 mM or 100 mM) were made by dissolving in water and stored on ice. CORM-2 stocks were kept in the dark and used within 1 h, while CORM-3 stocks were used fresh or on the following day after storage at 4 °C.

**Fig. 2:**
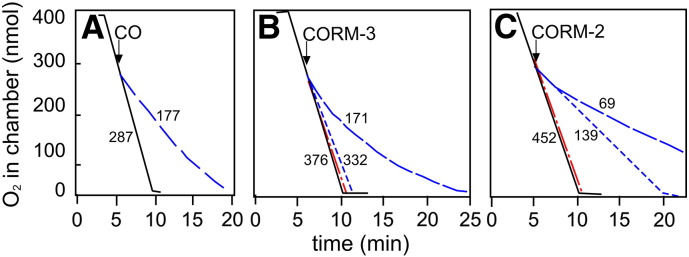
CO, CORM-3 and CORM-2 inhibit respiration in wild type *E. coli* membrane particles. Wild type *E. coli* membrane particles were added to an oxygen electrode chamber in buffer (2 ml) to a final concentration of approximately (A) 60 μg/ml, (B) 170 μg/ml, or (C) 100 μg/ml. The chamber was closed and respiration was initiated by the addition of 6.25 mM NADH. CO and CO-RMs were then added as indicated at approximately 75% of oxygen saturation (310 nmol O_2_ in the chamber). Traces show dissolved oxygen in the chamber as follows: (A) control (black solid line) and 100 μM CO (blue broken line); (B) control (black solid line), 100 μM iCORM-3 (red dot dash line), 100 μM CORM-3 (blue short dash line), 400 μM CORM-3 (blue long dash line); (C) control (black solid line), 100 μM RuCl_2_(DMSO)_4_ (red dot dash line), 50 μM CORM-2 (blue short dash line), 100 μM CORM-2 (blue long dash line). Respiration rates (nmol min^− 1^ mg^− 1^ protein) 2 min following the addition of CO-RM are shown adjacent to each trace. These data are representative of at least 3 technical and 2 biological replicates.

Two different control molecules were used. RuCl_2_(DMSO)_4_, used as a control for CORM-2, was supplied by Dr. Tony Johnson (Chemistry Department, The University of Sheffield) and stock solutions (10 mM) were made fresh each day by dissolving in water. The structure of this compound is shown in Fig. 1C [42]; this compound was used in Fig. 2. The second control compound, inactive iCORM-3 was prepared as previously described [15,43] by dissolving CORM-3 in phosphate-buffered saline (PBS), and bubbling with O_2_-free nitrogen (BOC, Guildford, GU2 5XY) at regular intervals over 2 h. After such time, little CO release can be detected (less than 5% than of the same concentration of CORM-3) in a myoglobin assay (see below). This process is thought to release labile CO, which escapes upon bubbling, leaving a CO-depleted iCORM-3 compound in solution [15]. However, growing evidence suggests that CORM-3 is unable to release large quantities of CO under these conditions, but rather the carbonyl compound is altered forming a stable species that releases CO more slowly [15,44–46]. This inactivation could produce a very slow CO-releasing tri-carbonyl complex [41] or a di-carbonyl compound, which is formed via the release of CO_2_ from CORM-3 [45]. It is acknowledged that iCORM-3 is an imperfect control, since it has not been exposed to biological molecules or intracellular species that may induce release of more CO and its structure is not known. However, iCORM-3 has been used as a control compound for CORM-3 by several research groups [15,45] and a recent paper [47] has revealed the transcriptomic response of *E. coli* to this compound, allowing further clarification as to which transcriptomic changes result from CO release from CO‐RMs, and which from the ruthenium compound devoid of labile CO. The advantage of iCORM-3 is that it is produced directly from the CORM-3 molecule and is therefore much more likely to mimic the compound present in vivo after CO has been released [47].

CO release from both CORM-3 and iCORM-3 to ferrous myoglobin was assayed as before [15,44] in a dual-wavelength scanning spectrophotometer [48]. Data were plotted as the difference between the spectrum of a CORM-3-reacted and reduced sample minus the spectrum of a reduced sample [49]. Where indicated, CO was added as a saturated solution made by bubbling from a cylinder (BOC, Guildford, GU2 5XY) at room temperature for 15 min.

### 2.4 Measurement of O_2_ consumption

*E. coli* membrane particles were suspended in sonication buffer (50 mM Tris–HCl, 2 mM MgCl_2_ and 1 mM EGTA, pH 7.4) in a stirred Perspex chamber fitted with a Clark-type polarographic O_2_ electrode (OXY041A, Rank Bros Ltd., Bottisham, CB25 9DA) held at 37 °C [50]. Both open and closed O_2_ electrode systems were used. In closed experiments, NADH (6.25 mM) was added to stimulate respiration and the CO-RM compound or CO-saturated solution was added when the dissolved oxygen tension in the chamber reached approximately 155 μM. In open electrode experiments, the chamber was initially closed and NADH (6.25 mM or 12.5 mM) was added to stimulate respiration. Compound(s) were added 1 min after the dissolved oxygen tension in the chamber reached anoxia and the lid removed 1 min later. This allowed continuous O_2_ diffusion from the vortex surface into the sample at a rate that is defined by K*_L_a*, the gas transfer coefficient from gas to liquid. This constant is dictated by the reaction volume, surface area and temperature [51], all of which were rigorously controlled and quantified. A typical K*_L_a* value, i.e. the rate constant for the half-time of oxygen diffusion into anoxic medium from the atmosphere, was 0.35 min^− 1^. The extent of respiratory inhibition was determined by measuring the time to oxygen re-accumulation after removal of the chamber lid, as previously described [52]. This method has the distinct advantage, as exploited before [52,53], that prolonged measurements can be made without exhaustion of oxygen.

### 2.5 UV–visible spectroscopy

Difference spectra (i.e. the difference between the spectrum of a CORM-3-reacted and reduced sample minus the spectrum of a reduced sample) of *E. coli* membrane particles suspended in sonication buffer (8–19 mg/ml) were recorded in a dual wavelength spectrophotometer [48] using a 10 mm path length cuvette. Membranes were reduced by the addition of a few grains of dithionite and then treated with CORM-3 (100 μM) or CO bubbling. Spectra were recorded in triplicate. Where appropriate, CORM-3 was pre-incubated for 5 min with NAC (1 mM) prior to addition to the membranes.

### 2.6 The deoxygenation of oxyleghaemoglobin by membranes containing only cytochrome *bd-*II

Determination of oxygen affinities was carried out as described previously by D'mello et al. [32]. Oxygenated soybean leghaemoglobin (kindly donated by Dr. C. Appleby) was diluted to 10–20 μM in phosphate buffer (50 mM, pH 7.4) containing 1 mM EDTA. Deoxygenation of the globin by respiration of the membranes was monitored using classical dual wavelength spectrophotometry as described by Kalnenieks et al. [48]; the measuring monochromater was set at 558 nm and the reference at 577 nm. The custom-made 1.3 ml capacity cuvette was filled with the globin solution and sealed with a finely perforated stopper, through which substrate solution (NADH, 3 mM) was injected. The stability of the oxygenated globin was checked by monitoring ΔA for 5–10 min. After addition of membranes, globin deoxygenation was continuously monitored by plotting ΔA_577–558_. Data were analysed as described previously by D'mello et al. [54]. Four separate determinations were carried out on each sample; means and standard deviations are given.

### 2.7 Ruthenium uptake by growing cells

Either CORM-3 (40 μM) alone or with NAC (400 μM) or CORM-2 (20 μM) alone or with NAC (200 μM) was added during logarithmic phase to aerobic cultures of *E. coli* grown in Evans medium [55]. This medium was used for this work to maintain consistency with other metal uptake analyses done within this laboratory [47]. Samples (20 ml) were taken before the addition of CORM-3 and at intervals thereafter. Cells were harvested by centrifugation at 5500 rpm for 5 min in polypropylene tubes (50 ml). Culture supernatants were retained for analysis. Cell pellets were washed three times in 0.5% HNO_3_ (0.5 ml each; Aristar nitric acid (69%, v/v)) to remove loosely bound elements. Supernatants collected from the washes were also retained for analysis. Samples were analysed using a Spectro Ciros^CCD^ (Spectro Analytical) inductively coupled plasma-atomic emission spectrometer (ICP-MS). The percentage of ruthenium recovered from these samples varied from 74 to 90% of the total added to the cultures. To calculate intracellular ruthenium concentrations, published values for individual cell dry mass and volume were used [56].

## 3 Results

### 3.1 CORM-2, CORM-3 and CO inhibit respiration in *E. coli* membrane particles

In many previous studies by others and ourselves, summarised in the Introduction, CO-RMs have been shown to be more effective inhibitors of growth and respiration than is CO administered as a solution of the gas (for example [17,18,21]). It is thought that this may be due in part to an accumulation of CORM-3 inside bacterial cells leading to high CO concentrations at the target site(s) [18]. CORM-3 releases 1 mol of CO for each mole of CO-RM [15] whereas CORM-2 releases 0.7 mol of CO for each mole of compound [40] and these stoichiometries are unchanged by factors such as sulfite species that change only the rate of CO release [47]. We investigated the effects of CORM-2, CORM-3 and CO gas on respiration in membrane particles prepared from wild type *E. coli*. Unlike previous studies with intact bacteria, CO (100 μM) significantly inhibited respiration of membrane particles (by 38% at 2 min following the addition of CO), and to a greater extent than 100 μM CORM-3 (12%) (Fig. 2A and B).

CORM-2 was found to be more inhibitory to respiration than CORM-3; 100 μM CORM-2 inhibited respiration of wild type *E. coli* membrane particles by 85% (Fig. 2C). In contrast, 400 μM CORM-3 inhibited respiration by only 55% of the control rate, as measured 2 min following CORM-3 addition. This is in agreement with the findings of Nobre et al. [17] who found that higher concentrations of CORM-3 than CORM-2 were required to decrease viability in *E. coli* and *S. aureus* cultures. Importantly, the control compounds iCORM-3 and RuCl_2_(DMSO)_4_ had no significant inhibitory effects on respiration: 100 μM iCORM-3 inhibited respiration by 6% of the control rate, while addition of 100 μM RuCl_2_(DMSO)_4_ had no effect on the respiration rate.

### 3.2 Cells expressing only cytochrome *bd*-I as sole oxidase are least susceptible to inhibition by CORM-3

**Fig. 3:**
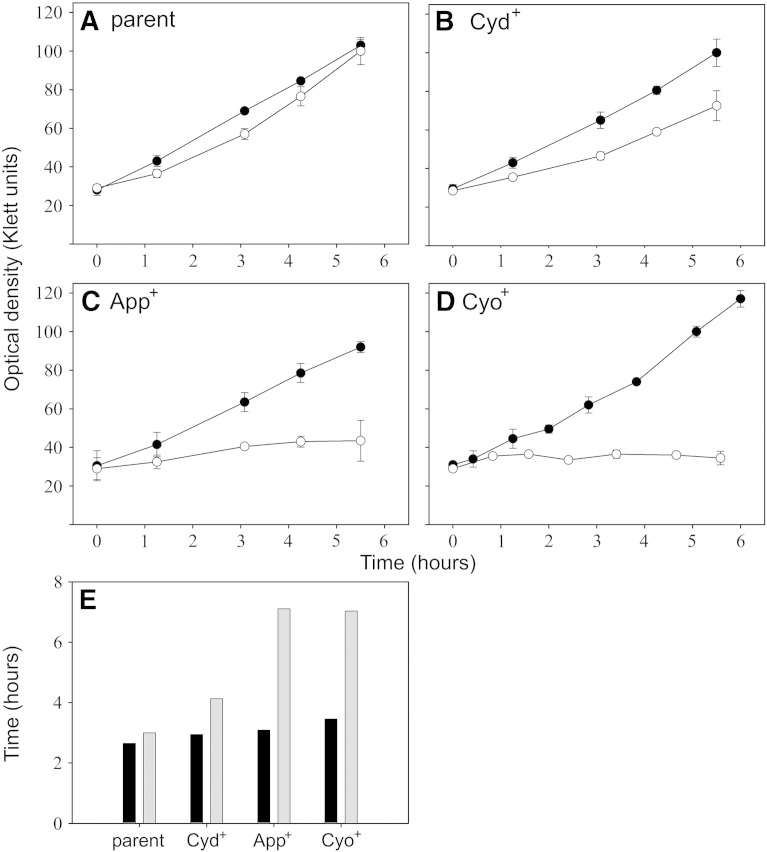
A strain containing cytochrome *bd-*I as sole oxidase is more resistant to growth inhibition by CORM-3 than are strains expressing other oxidases. CORM-3 (12.5 μM) was added at early exponential phase to cultures of *E. coli* as follows: (A) parent strain; (B) expressing cytochrome *bd-*I only; (C) expressing cytochrome *bd-*II only and (D) expressing cytochrome *bo′* only. Data in (A)–(D) show growth in the absence of any addition (closed symbols) and presence (open symbols) of CORM-3. Points are means and standard deviations of three biological replicates. (E) Shows the doubling times of these strains in the 2 h following CORM-3 addition, before (black bars) and after (grey bars) the addition of CORM-3. These data are representative of three biological replicates.

Inhibition of respiration by CO from CORM-3 is thought to be one of the major mechanisms of killing by the metal carbonyl compound [18,21,22], but is probably not wholly responsible for the toxic effects. Previous work has confirmed that CO from CORM-3 is internalised by *E. coli* cells and binds to the terminal oxidases of the aerobic respiratory chain [18]. In order to understand better the mechanism of respiratory inhibition by CORM-3, and because most bacteria (unlike mitochondria) possess numerous distinct oxidases, we asked whether mutants expressing each of the three oxidases of *E. coli* differ in their sensitivity to this well-characterised, water-soluble CO-RM and the CO released from it. Growth of mutants was assessed in the presence of 12.5 μM CORM-3 (Fig. 3). The levels of oxidases present in these strains are those resulting from the natural promoter strengths without use of inducers; precise control or standardisation of levels of oxidase expression is impractical. The strain containing cytochrome *bd*-I only was found to be most resistant to growth inhibition in the presence of CORM-3 (Fig. 3B, E) and the strains expressing cytochromes *bd*-II or *bo′* as the only oxidase were found to be most sensitive (Fig. 3C, D, and E). The relative resistance to CORM-3 of the strain containing only cytochrome *bd*-I is reminiscent of the resistance of this oxidase to NO [57], cyanide [58] and other inhibitors [59].

In light of this finding, membrane particles prepared from these single oxidase-expressing *E. coli* mutants were assessed for their susceptibility to respiratory inhibition by CORM-3. In this experiment, in view of the slow and incomplete inhibition of respiration (Fig. 2), we used an open electrode experimental design in which inhibition can be monitored over an hour or more, as before [21]. An oxygen electrode was used to monitor the dissolved oxygen concentration in a sample of *E. coli* membrane particles, which were stimulated to respire by the addition of NADH. Following the complete depletion of oxygen from the sample, CORM-3 was added and the lid of the chamber removed allowing oxygen to enter the system. The extent of respiratory inhibition was determined by measuring the time taken for oxygen re-accumulation to begin after removal of the chamber lid, in the presence of CORM-3. The respiration of a more sensitive strain will be inhibited to a greater extent, leading to a shorter time to reoxygenation [52,53].

**Fig. 4:**
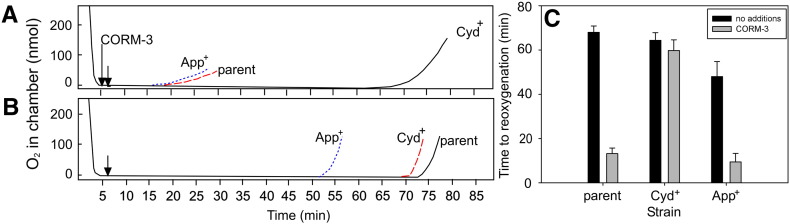
Cytochrome *bd*-I is resistant to respiratory inhibition by CORM-3. Membranes prepared from mutants containing only one of the three terminal oxidases of *E. coli* were added to an oxygen electrode chamber in sonication buffer (2 ml) to a final concentration of approximately 370 μg/ml and stimulated to respire by the addition of NADH (12.5 mM). The point at which CORM-3 (100 μM) was added is indicated by the first, longer arrow in (A). The control traces, in which no CORM-3 was added are shown in (B). The lid was removed from the chamber 1 min later, indicated by the second arrow. The traces show dissolved oxygen in the chamber and indicate the times taken for the chamber to begin to reoxygenate for the strains containing cytochrome *bd*-I only (black lines), cytochrome *bd*-II only (blue dotted lines) and the parent strain (red dashed lines). These data are from one representative experiment. (C) Shows the time to reoxygenation for each strain with CORM-3 (grey bars) or no additions (black bars). These data are the means and standard deviations of at least 3 technical repeats.

When respiring membrane particles containing either cytochrome *bd*-II (App^+^) or all three oxidases (parent strain) were treated with 100 μM CORM-3, the time taken for the chamber to reoxygenate was significantly reduced (by 80–82%) (Fig. 4A) compared to when no compound was added (Fig. 4B). Thus, CORM-3 significantly inhibits respiration in these strains. However, when membrane particles prepared from a strain expressing only cytochrome *bd*-I (Cyd^+^) were treated with 100 μM CORM-3, the time to reoxygenation of the electrode chamber was reduced by only 7% compared to that when no compound was added. This confirms that membranes possessing only cytochrome *bd*-I are the most resistant to CORM-3. These results are summarised in Fig. 4C. Attempts were made to perform this experiment using membranes expressing cytochrome *bo′* as the only terminal oxidase; however, due to the much slower respiration rate of this strain, it was not possible for the chamber to maintain an oxygen tension of 0% for an extended period of time in the control experiment in which no compound was added.

**Fig. 5:**
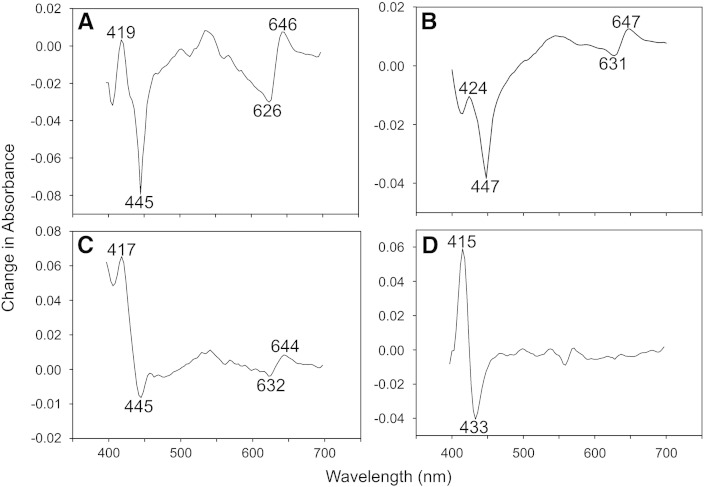
Difference spectra of membrane particles expressing only a single oxidase. Membranes were prepared from *E. coli* respiratory mutants and diluted with buffer to a final protein concentration of 8–19 mg/ml. Spectra were obtained 10 min after the addition of CORM-3 to membranes from the following strains: (A) wild type; (B) cytochrome *bd*-I only; (C) cytochrome *bd*-II only; (D) cytochrome *bo′* only. Data were plotted using a scanning dual beam spectrophotometer as the difference between the spectrum of a dithionite reduced sample incubated with 100 μM CORM-3 minus the spectrum of a reduced sample. Data have been smoothed in Sigma plot graphing software with a sampling proportion of 0.1 and a polynomial degree of 9.

The oxidase composition of the respiratory mutants used in this work was confirmed by identification of the CO-reactive cytochromes (Fig. 5). Here we show CORM-3 difference spectra (i.e. the difference between the spectrum of a CORM-3-reacted and reduced sample minus the spectrum of a reduced sample) of membrane particles prepared from the single oxidase-expressing strains. In Fig. 5A–C, the Soret feature consisting of a peak near 420 nm (417–424 nm) and the trough at 445–447 nm demonstrate the binding of CO from CORM-3 to the *bd*-type oxidase [60,61]. In the case of Cyo^+^ membranes (Fig. 5D), the presence of cytochrome *o′* is revealed by a markedly blue-shifted absorbance minimum at 433 nm (due to loss of the ferrous cytochrome *o*′) accompanied by a 415 nm peak (due to formation of the Fe(II)–CO adduct) [62]. The signals in the near-red region are more informative; the peak at 644–646 nm is diagnostic of the binding of CO to cytochrome *d* exclusively, whilst the minimum at 626–632 is due to loss of absorbance of the Fe(II) form of haem *d* (Fig. 5A–C). Cytochrome *bo′* does not exhibit bands in this region and, accordingly, the CORM-3 difference spectrum of Cyo^+^ membranes (Fig. 5D) is featureless beyond about 580 nm.

### 3.3 Determination of the oxygen affinity of cytochrome *bd*-II in membranes by deoxygenation of oxyleghaemoglobin

Several authors report the oxygen affinities of cytochromes *bo′* and *bd*-I in *E. coli* [63–65] but most of these rely on the use of membrane-covered oxygen electrodes, which cannot provide the sensitivity required for enzymes with such high affinities. However, we have previously used the deoxygenation of oxymyoglobin or oxyleghaemoglobin devised by Bergersen and Turner [66] to measure reliably the affinities of each of these oxidases in situ [32,67]. Using this method, we determined the *K*_m_ of *E. coli* membrane particles containing cytochrome *bo′* as the only terminal oxidase to be 0.2 μM and 0.46 μM with each globin respectively [67], whereas *E. coli* membrane particles containing only cytochrome *bd* had much higher oxygen affinity, with a *K*_m_ of 5.8 nM determined using leghaemoglobin [32].

**Fig. 6:**
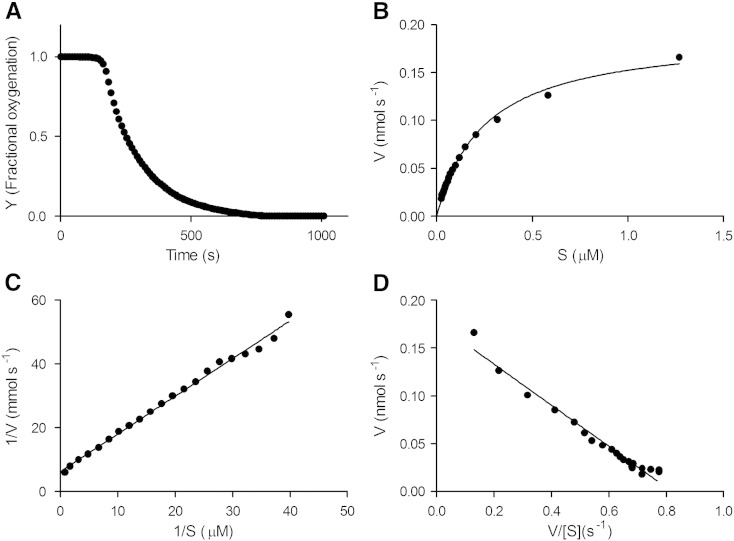
Determination of oxygen affinity of cytochrome *bd*-II in membranes by the deoxygenation of oxyleghaemoglobin. (A) Deoxygenation of oxyleghaemoglobin during respiration of membranes containing cytochrome *bd*-II as the only oxidase (reaction stimulated with NADH, 3 mM final concentration). (B) Oxygen consumption rates (V) and oxygen concentrations (S) were derived from the Appleby and Bergersen [77] equations. (C) Lineweaver–Burk plot and (D) Eadie-Hofstee plot. The affinity of cytochrome *bd*-II for oxygen (*K*_m_(O_2_)) was determined to be 0.24 μM (SD 0.019), with a *V*_max_ value of ~ 0.2 nmol s^− 1^ mg protein^− 1^ (SD 0.02).

Here we report the application of a similar method to obtain for the first time the oxygen affinity of cytochrome *bd*-II in preparations that contain only this oxidase. Fig. 6 shows the deoxygenation kinetics of oxyleghaemoglobin by *E. coli* membranes expressing cytochrome *bd*-II. Precautions were taken to ensure (i) that the dual-wavelength absorbance measurements at the selected wavelengths were unaffected by turbidity of the preparation, (ii) that the measured affinities were independent of membrane concentration and (iii) that the observed absorbance changes were due to deoxygenation, not globin oxidation, as described previously [32]. The progress of deoxygenation was used to calculate the fractional oxygenation of leghaemoglobin (Fig. 6A), from which we computed a plot of oxidase velocity versus oxygen concentration (Fig. 6B) and thence Lineweaver–Burk (Fig. 6C) and Eadie–Hofstee (Fig. 6D) plots. The affinity of cytochrome *bd*-II for oxygen (*K*_m_(O_2_)) was determined to be 0.24 μM (SD 0.019), with a *V*_max_ value of ~ 0.2 nmol s^− 1^ mg protein^− 1^ (SD 0.02). The *K*_m_(O_2_) for cytochrome *bd*-II calculated in this work is quoted in Table 1 alongside literature values of the oxygen affinity of other bacterial oxidases calculated by this method.

### 3.4 NAC prevents the inhibitory effects of CO-RMs on respiration, but does not prevent binding of CO from CORM-3 to the terminal oxidases of *E. coli*

**Fig. 7:**
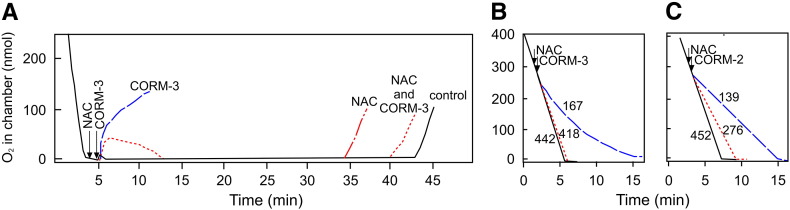
NAC prevents CORM-2 and CORM-3-dependent inhibition of respiration in *E. coli* membrane particles. Wild type *E. coli* membrane particles were added to sonication buffer (2 ml) to a final concentration of approximately (A) 1490 μg/ml, (B) 70 μg/ml or (C) 100 μg/ml and respiration was initiated by the addition of NADH (6.25 mM). In (A), the lid was removed from the chamber 1 min after the addition of CORM-3; arrows indicate the addition of NAC or CORM-3 (both at 400 μM); the traces show dissolved oxygen in the open chamber for uninhibited respiration (black solid lines) or oxygen consumption in the presence of CO-RM (blue dashed lines), CO-RM and NAC (red dotted lines) or NAC alone (red dot dash line). The time taken for the chamber to reoxygenate (given in min in parentheses) was measured for the various conditions: with no additions (35); with CORM-3 (0); with NAC and CORM-3 (33); with NAC alone (28). In (B) and (C), a conventional closed oxygen electrode apparatus was used, uninhibited respiration is indicated by the black solid lines, oxygen consumption in the presence of CO-RM by the blue dashed lines and that in the presence of CO-RM and NAC by the red dotted lines. In (B), arrows indicate the addition of NAC or CORM-3 (both at 400 μM). In (C), arrows indicate the addition of NAC (500 μM) or CORM-2 (50 μM). In (B) and (C), respiration rates (nmol min^− 1^ mg^− 1^ protein) 2 min following the addition of (B) CORM-3 and (C), CORM-2 are shown on adjacent to each trace. These data are representative of at least 3 technical and 2 biological replicates.

It is well documented that thiol-containing compounds such as NAC prevent metal-containing CO-RMs from exerting their antibacterial effects [22,31,46]. In particular, Desmard et al. [22] reported that NAC prevents the inhibition of respiration by CORM-3 in *P. aeruginosa* cultures. Here we report that NAC prevents the inhibition of respiration by both CORM-2 and CORM-3 in membrane particles prepared from wild type *E. coli* (Fig. 7). Fig. 7A shows an open oxygen electrode experiment in which the time taken for the electrode chamber to reoxygenate is measured in the presence of respiring wild-type *E. coli* membrane particles and, where indicated, with CORM-3 in the presence or absence of NAC. The addition of CORM-3 (400 μM) caused the chamber to reoxygenate immediately, indicating that respiration was inhibited (Fig. 7A, blue dashed line). However, when NAC (400 μM) was added prior to CORM-3, respiration was protected so that the time taken for the chamber to reoxygenate was restored to 87% of the control time in which no inhibitor was added. NAC was added to the chamber in the absence of CORM-3 and found to cause reoxygenation in 74% of the time taken under control conditions.

Closed oxygen electrode measurements confirmed the finding that 100 μM NAC protects *E. coli* membrane particles from respiratory inhibition by the same concentration of CORM-3 (Fig. 7B). The respiration rate of membrane particles 2 min after the addition of CORM-3 (400 μM) was 167 nmol min^− 1^ mg^− 1^ compared to 442 nmol min^− 1^ mg^− 1^ in the absence of inhibitor and 418 nmol min^− 1^ mg^− 1^ in the presence of CORM-3 and NAC. Furthermore, NAC also reduces respiratory inhibition of *E. coli* membrane particles by CORM-2, but here a 10-fold excess of NAC is needed in order to reduce the inhibitory effect of CORM-2 by approximately 50% (Fig. 7C). The respiration rate of membrane particles 2 min after the addition of CORM-2 (50 μM) was 139 nmol min^− 1^ mg^− 1^ compared to 452 nmol min^− 1^ mg^− 1^ in the absence of inhibitor and 276 nmol min^− 1^ mg^− 1^ in the presence of CORM-2 and NAC.

**Fig. 8:**
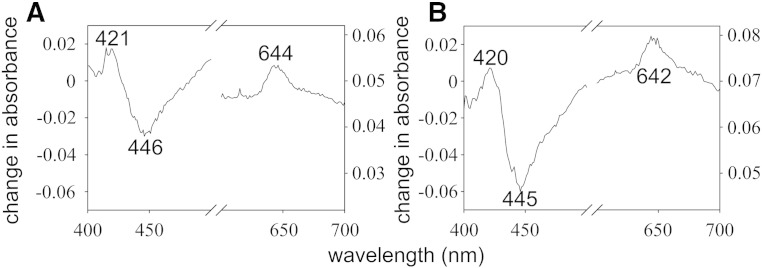
Effect of NAC on the reaction of the terminal oxidases in wild type *E. coli* membrane particles with CORM-3. Wild type *E. coli* membrane particles were added to sonication buffer to a final concentration of approximately 10 mg/ml and incubated with (A) CORM-3 (100 μM) and (B) CORM-3 (100 μM) pre-incubated for 5 min with NAC (1 mM). Spectra were obtained 5 min after the addition of CORM-3 to membranes using a scanning dual beam spectrophotometer. Data were plotted as the difference between the spectrum of a dithionite reduced sample incubated with CORM-3 minus the spectrum of a reduced sample. Data have been smoothed in Sigma plot graphing software with a sampling proportion of 0.1 and a polynomial degree of 9.

We considered the possibility that NAC prevented the interaction between the terminal oxidases and CO from CORM-3. Difference spectra (i.e. the difference between the spectrum of a CORM-3-reacted and reduced sample minus the spectrum of a reduced sample) were collected in both the absence (Fig. 8A) and presence (Fig. 8B) of a 10-fold excess of NAC and found to have a peak at approximately 420 nm and a trough at approximately 446 nm in addition to a peak at approximately 644 nm. As in Fig. 5, the last is diagnostic of the CO-bound cytochrome *d* of *E. coli* [49], while the Soret features are attributed to CO binding to haems *d* and *b*_595_. Importantly, the intensity of these features was not significantly altered in the presence of NAC (Fig. 8B), confirming that this compound does not interfere with the binding of CO released from CORM-3 with the terminal oxidases of *E. coli*. This concurs with the findings of Desmard et al. [46] who concluded that, as the activities of different CO-RMs are affected differently by thiol compounds, it is unlikely that these compounds have downstream effects such as affecting the interaction of CO with cytochromes.

### 3.5 The antioxidant properties of NAC do not abolish the inhibition of respiration by CORM-3

**Fig. 9:**
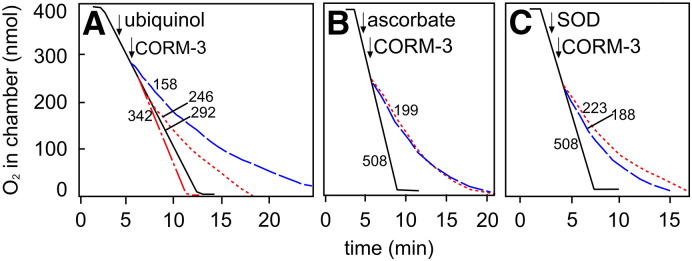
Antioxidants and superoxide dismutase (SOD) do not prevent CORM-3-dependent inhibition of respiration to the same extent as thiol compounds. Wild type *E. coli* membrane particles were added to the oxygen electrode in sonication buffer (2 ml) to a final concentration of approximately 60 μg/ml in (A) and 100 μg/ml in (B). The chamber was closed and respiration was initiated by the addition of 6.25 mM NADH. The first arrows in each panel indicate the addition of the antioxidant or enzyme: (A) ubiquinol (100 μM); (B) ascorbate (1 mM); (C) SOD (250 units) to the chamber, while the second arrows indicate the addition of CORM-3 (400 μΜ). The black solid lines show the uninhibited respiration rate, the blue dashed lines show oxygen consumption in the presence of CORM-3 and the red dotted lines show oxygen consumption in the presence of CORM-3 and the antioxidant (A and B) or SOD (C). In (A), the red dot dash line shows oxygen consumption in the presence of ubiquinol alone. Respiration rates (nmol min^− 1^ mg^− 1^ protein) 2 min following the addition of CO-RM are shown adjacent to each trace. Traces are representative of 2 biological replicates, each with 3 technical replicates.

It has been proposed that NAC prevents the effects of CORM-3 by virtue of its antioxidant properties [31]. To explore this hypothesis, we investigated whether other antioxidants that do not contain thiol groups were able to prevent the inhibition of respiration by CORM-3. Ubiquinol (200 μM) (Fig. 9A) was able to decrease the inhibition of respiration by 400 μM CORM-3 (by 36% compared to the rate 2 min after the addition of CORM-3), but not to the same extent as NAC (400 μM, which caused a 60% decrease in inhibition compared to the rate 2 min after the addition of CORM-3) (Fig. 7B). When ubiquinol was added to the oxygen electrode chamber in the absence of CORM-3, respiration was stimulated slightly, which is consistent with the role of this compound in carrying electrons to the terminal oxidases and could account to some extent for the decreased inhibition when ubiquinol and CORM-3 were added simultaneously. Another antioxidant, ascorbate (1 mM) caused no reduction in the inhibition of respiration by CORM-3 (Fig. 9B). Ubiquinol and ascorbate both have a redox potential of + 60 mV [68], and therefore their different effects on the ability of CORM-3 to inhibit respiration are unlikely to be explained by this property. Finally, superoxide dismutase (SOD, 250 units) was added to the chamber prior to the addition of CORM-3, as it was expected that, if superoxide were generated by the inhibition of respiration by CORM-3, SOD would convert it to oxygen, which would be seen as an upward deflection of the oxygen electrode trace [69]; however this was not seen (Fig. 9C), suggesting that biologically relevant concentrations of superoxide do not accumulate when respiration is inhibited by CORM-3. In addition, the presence of SOD did not significantly reduce the degree of inhibition experienced by *E. coli* membrane particles treated with CORM-3.

### 3.6 NAC prevents the uptake of CORM-2 and CORM-3 into bacterial cells

**Fig. 10:**
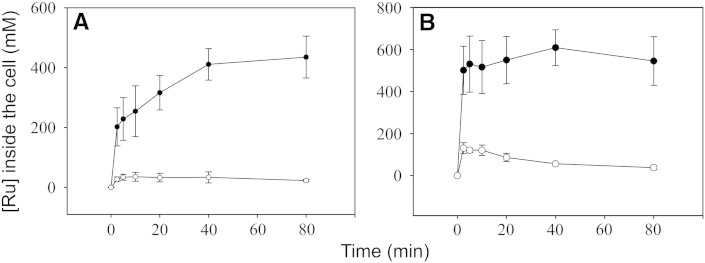
NAC significantly reduces the uptake of CORM-2 and CORM-3 into bacterial cells. Cultures of wild type *E. coli* were grown to mid log phase (OD_600_ ~ 0.5) prior to the removal of 20 ml samples both before, and at regular intervals after, the addition of CO-RM. In (A) Ru uptake as CORM-3 (40 μM) in the absence (closed symbols) or presence (open symbols) of 400 μM NAC is shown. In (B) Ru uptake as CORM-2 (20 μM) in the absence (closed symbols) or presence (open symbols) of 200 μM NAC is shown. Cell pellets were assayed for Ru content by inductively coupled plasma mass spectrometry. Data are the means and standard deviations of 3 biological replicates.

Since reaction of CORM-3-derived CO with the terminal oxidases in membranes is not prevented by NAC (Fig. 8), we tested the hypothesis that NAC reduces the uptake of CORM-2 and CORM-3 by *E. coli* by measuring intracellular ruthenium content at various time points after treatment with either the CO-RM alone, or the CO‐RM in the presence of a 10-fold excess of NAC. Following treatment of cultures of *E. coli* with sub-lethal concentrations of CORM-3 (40 μM; Fig. 10A) or CORM-2 (20 μM; Fig. 10B), ruthenium rapidly accumulates to approximately 8 and 30 times the concentration outside the cell respectively. However, the uptake of these CO-RMs by wild type *E. coli* cells was dramatically reduced by NAC, by approximately 8-fold for CORM-3 (Fig. 10A) and approximately 5-fold for CORM-2 (Fig. 10B).

## 4 Discussion

Here, we explore the relative sensitivities of strains expressing each of the three quinol oxidase protein complexes of *E. coli* to inhibition by CO and CO-RMs. The latter are finding increasing application in physiological studies of higher organisms and CO-RMs are being considered as potential antimicrobial agents. It is becoming clear that CO gas and CO-RMs have different modes of action against many bacterial processes; therefore, although there is an extensive literature on the interactions of cytochromes *bd*-I and *bo′* (but not *bd*-II) with CO gas and other ligands, it cannot be assumed that oxidases will respond similarly to CO-RMs. It seems likely that the bactericidal effects of CO-RMs are attributable to interaction with cellular processes and molecules that are distinct from those targeted by antibiotics, so that the use of CO-RMs has considerable merit in therapies. In this paper, we use CORM-3 for most respiration measurements, since this is a water-soluble CO-RM that has been extensively characterised by others and ourselves (for references, see Introduction).

Previous transcriptomic data with *E. coli* [18] demonstrated the down-regulation by CORM-3 of the *cyo* genes encoding cytochrome *bo′* and modest up-regulation of the *cydAB* genes encoding cytochrome *bd*-I; the effects on the *appBC* genes encoding cytochrome *bd*-II were not revealed. Further analyses demonstrated that the transcription of *appBC* is unaltered after addition for CORM-3 over 80 min, whereas expression of the *cydAB* genes was increased initially, followed by a decrease (~ 3-fold down). This might suggest a role for cytochrome *bd*-I in resisting CORM-3. The *cyo* genes appeared to be most responsive to CORM-3 treatment with a fast and sustained down-regulation (as low as ~ 50-fold decrease) over 80 min [47]. Here we show that a strain expressing only cytochrome *bd*-I is more resistant to CORM-3 than are strains expressing only cytochromes *bo′* or *bd*-II. Cytochrome *bd*-I is also reported to protect the cell from various environmental stresses such as cyanide, azide and divalent metal ions [59] and cytochrome *bd*-I null mutants are more sensitive to hydrogen peroxide than are wild type cultures [70].

Significantly, cytochrome *bd*-I has been shown to protect *E. coli* from NO-induced growth inhibition [57]. Explanations have been sought in the ligand-binding kinetics of cytochromes *bd*-I and *bo′*. The *K*_d_ (NO) is 4.4 nM for cytochrome *bo′* but 0.55 nM for cytochrome *bd* [57]. However, the basis of the relative insensitivity of cytochrome *bd* to NO appears to be the fast *k*_off_ (0.163 s^− 1^) [57], 5-fold faster than that observed with NO for cytochrome *bo′* (0.03 s^− 1^) and indeed almost all haem proteins. Interestingly, NO also up-regulates *cydAB* expression [71], as does (slightly) CO [18].

In the case of CO, the apparent *K*_d_ value for cytochrome *bd* in membranes appears to be ~ 70 nM (based on a method that involves observing the distribution of CO between the oxidase and myoglobin) [61] compared to 1.7 μM for cytochrome *bo′* [62]. For cytochrome *bd*, the *k*_off_ is 6 or 1.6 s^− 1^ according to the method used [72]. The experimentally determined *K*_d_ is consistent with the faster ‘off ‘rate of 6 s^− 1^ [61]. Seeking an explanation of the CO‐RM data by comparing the *k*_off_ for CO from cytochromes *bd* and *bo′* is complicated by literature disagreements. Cheesman et al. [62] report a *K*_off_ (CO) value of ‘< 10 s^− 1^’ for cytochrome *bo′*, but the dependence of the pseudo-first-order rate upon CO concentration suggests a value around 1 s^− 1^. Subsequent citing of these data, however, gives a *K*_off_ (CO) value of 0.1 s^− 1^[73].

In summary, whereas the insensitivity of cytochrome *bd* to NO appears to be explained by the fast *k*_off_, no simple analysis of the ‘off rates’ for CO from the two oxidases is possible. If we accept a *k*_off_ of 6 s^− 1^ for cytochrome *bd* [72] then the published data for cytochrome *bo′* are either very similar (< 10 s^− 1^) [62] or significantly lower (0.1 s^− 1^) [73] and the differential sensitivity to CORM-3 is not readily explained by their reactivities with CO gas alone. The affinity of cytochrome *bd*-II for CO has not been reported. These difficulties support our contention (see Introduction) that sensitivity of cellular processes to CO-RMs cannot be predicted solely on the basis of the response to CO gas.

Further distinction between the cellular roles of cytochromes *bd*-I and *bd*-II is evident from consideration of affinities for oxygen. The former has long been considered to have an exceptionally high affinity, with *K_m_* values as low as 3–8 nM being reported for respiring cells and membranes of *E. coli* in which cytochrome *bd*-I was the dominant oxidase [32] (Table 1). However, the near identity of the spectral signatures of cytochromes *bd*-I and *bd*-II [60] does not allow reliable conclusions to be drawn about the oxygen affinity of the latter. Measurements of the *K_m_* of cytochrome *bd*-II for oxygen were published recently [37] but were performed using a membrane-covered Clark electrode with inadequate sensitivity in the nanomolar range. The value cited for cytochrome *bd*-II (2 μM) therein was almost 10-fold higher than the value reported in the present work. Furthermore, the *K_m_* for *bd*-I was almost 100-fold higher than previous determinations using the globin deoxygenation method [32] (Table 1). Both papers agree, however, on the much higher oxygen affinity of cytochrome *bd*-I. The values published for assays that have used the sensitive globin deoxygenation method are summarised in Table 1. The *K*_m_(O_2_) for *E. coli* cytochrome *bd*-II is clearly much higher than those reported for *E. coli* cytochrome *bd*-I and several other oxidase classes. The function of cytochrome *bd*-II in *E. coli* is currently unknown.

We attribute the different susceptibilities of whole cells [21] and membrane particles as reported in the current work, to the ability of CO gas to access the terminal oxidases of the respiratory chain more easily in membrane particles. In addition, it is known that CO release from CORM-3 is markedly promoted in the presence of compounds such as dithionite or sulfite [44]. We have suggested that intracellular compounds like sulfite might promote CO release from CORM-3 after internalisation, which may explain why higher concentrations of CORM-3 are required in order to observe inhibition of respiration in membrane particles.

In the present work, we have achieved increased understanding of the striking ability of NAC to abrogate the inhibitory effects of CO-RMs. Because the literature shows thiol compounds to be effective in reducing the toxicity of several metal-containing CO-RMs [22,31,46], both CORM-2 and CORM-3 were used for this part of the present work. Here we show (i) that the antioxidant activity of NAC is not key to this property, (ii) NAC does not prevent CO release to terminal oxidase targets, and (iii) that uptake of CORM-2 and CORM-3 is severely inhibited by the presence of this compound. This reduced uptake is likely to contribute to the efficacy of NAC against the activity of some CO-RMs in a number of microbial and cell culture studies. It is interesting that in the presence of NAC and CORM-3, ruthenium accumulates inside the cell to only approximately the same concentration that is present outside the cell. A possible explanation for this is that NAC reacts with CORM-3 rendering it unable to be transported against a concentration gradient into the bacterial cell under these conditions. However we know nothing about how CO-RMs are transported and release CO inside cells (the “Trojan Horse” [21]). Our transcriptomic study of the effects of CORM-3 has revealed in *E. coli* dramatic up-regulation of the *mdtABC* genes, encoding an RND-family multidrug efflux pump, and *spy*, encoding a membrane-stress-responsive protein [18,47]. This implicates a transport response to CORM-3, different from the bacterial response to CO gas, and a clue to the bactericidal effectiveness of CORM-3. In the absence of any molecular information on the mechanism(s) of CO-RM uptake by cells, further comments can be only speculative.

## Figures and Tables

**Table 1:** Oxygen affinities of selected bacterial terminal oxidases as determined by the deoxygenation of globins.

Bacterium and oxidase	*K*_m_(O_2_) (μM)	Reference
*E. coli* cytochrome *bd-*II	0.24	This work
*E. coli* cytochrome *bd-*I	0.003–0.008	[32]
*Azotobacter vinelandii* cytochrome *bd*	4.5	[54]
*Klebsiella pneumoniae* cytochrome *bd*	0.02	[74]
*Campylobacter jejuni* cytochrome *bd* (*cio* or cyanide insensitive oxidase)	0.8	[75]
*E. coli* cytochrome *bo′*	0.016–0.35	[67]
*Campylobacter jejuni* cytochrome *cb’*	0.04	[75]
*Bradyrhizobium japonicum cbb_3_*-type oxidase	0.007	[76]

## References

[1] Keilin D. (1966). The History of Cell Respiration and Cytochrome.

[2] Marks G.S., Brien J.F., Nakatsu K., McLaughlin B.E. (1991). Does carbon monoxide have a physiological function?. Trends Pharmacol. Sci..

[3] Motterlini R., Otterbein L.E. (2010). The therapeutic potential of carbon monoxide. Nat. Rev. Drug Discov..

[4] Verma A., Hirsch D.J., Glatt C.E., Ronnett G.V., Snyder S.H. (1993). Carbon-monoxide — a putative neural messenger. Science.

[5] Furchgott R.F., Jothianandan D. (1991). Endothelium-dependent and endothelium-independent vasodilation involving cyclic-GMP — relaxation induced by nitric-oxide, carbon-monoxide and light. Blood Vessels.

[6] Boczkowski J., Poderoso J.J., Motterlini R. (2006). CO-metal interaction: vital signaling from a lethal gas. Trends Biochem. Sci..

[7] Bathoorn E., Slebos D.J., Postma D.S., Koeter G.H., van Oosterhout A.J., van der Toorn M., Boezen H.M., Kerstjens H.A. (2007). Anti-inflammatory effects of inhaled carbon monoxide in patients with COPD: a pilot study. Eur. Respir. J..

[8] Mann B.E. (2012). CO-releasing molecules: a personal view. Organometallics.

[9] Motterlini R., Mann B.E., Johnson T.R., Clark J.E., Foresti R., Green C.J. (2003). Bioactivity and pharmacological actions of carbon monoxide-releasing molecules. Curr. Pharm. Des..

[10] Motterlini R., Sawle P., Hammad J., Bains S., Alberto R., Foresti R., Green C.J. (2005). CORM-A1: a new pharmacologically active carbon monoxide-releasing molecule. FASEB J..

[11] Foresti R., Hammad J., Clark J.E., Johnson T.R., Mann B.E., Friebe A., Green C.J., Motterlini R. (2004). Vasoactive properties of CORM-3, a novel water-soluble carbon monoxide-releasing molecule. Br. J. Pharmacol..

[12] Koneru P., Leffler C.W. (2004). Role of cGMP in carbon monoxide-induced cerebral vasodilation in piglets. Am. J. Physiol. Heart Circ. Physiol..

[13] Sawle P., Foresti R., Mann B.E., Johnson T.R., Green C.J., Motterlini R. (2005). Carbon monoxide-releasing molecules (CO-RMs) attenuate the inflammatory response elicited by lipopolysaccharide in RAW264.7 murine macrophages. Br. J. Pharmacol..

[14] Alcaraz M.J., Guillen M.I., Ferrandiz M.L., Megias J., Motterlini R. (2008). Carbon monoxide-releasing molecules: a pharmacological expedient to counteract inflammation. Curr. Pharm. Des..

[15] Clark J.E., Naughton P., Shurey S., Green C.J., Johnson T.R., Mann B.E., Foresti R., Motterlini R. (2003). Cardioprotective actions by a water-soluble carbon monoxide-releasing molecule. Circ. Res..

[16] Sato K., Balla J., Otterbein L.E., Smith R.N., Brouard S., Lin Y., Csizmadia E., Sevigny J., Robson S.C., Vercellotti G., Choi A.M., Bach F.H., Soares M.P. (2001). Carbon monoxide generated by heme oxygenase-1 suppresses the rejection of mouse-to-rat cardiac transplants. J. Immunol..

[17] Nobre L.S., Seixas J.D., Romao C.C., Saraiva L.M. (2007). Antimicrobial action of carbon monoxide-releasing compounds. Antimicrob. Agents Chemother..

[18] Davidge K.S., Sanguinetti G., Yee C.H., Cox A.G., McLeod C.W., Monk C.E., Mann B.E., Motterlini R., Poole R.K. (2009). Carbon monoxide-releasing antibacterial molecules target respiration and global transcriptional regulators. J. Biol. Chem..

[19] Poole R.K., Lloyd D., Kemp R.B. (1973). Respiratory oscillations and heat evolution in synchronously dividing cultures of fission yeast Schizosaccharomyces-Pombe 972h. J. Gen. Microbiol..

[20] Reeder B.J., Svistunenko D.A., Wilson M.T. (2011). Lipid binding to cytoglobin leads to a change in haem co-ordination: a role for cytoglobin in lipid signalling of oxidative stress. Biochem. J..

[21] Wilson J.L., Jesse H.E., Hughes B.M., Lund V., Naylor K., Davidge K.S., Cook G.M., Mann B.E., Poole R.K. (2012). Ru(CO)3Cl(glycinate) (CORM-3): a CO-releasing molecule with broad-spectrum antimicrobial and photosensitive activities against respiration and cation transport in Escherichia coli. Antioxid. Redox Signal..

[22] Desmard M., Davidge K.S., Bouvet O., Morin D., Roux D., Foresti R., Ricard J.D., Denamur E., Poole R.K., Montravers P., Motterlini R., Boczkowski J. (2009). A carbon monoxide-releasing molecule (CORM-3) exerts bactericidal activity against *Pseudomonas aeruginosa* and improves survival in an animal model of bacteraemia. FASEB J..

[23] Wang R., Wu L.Y. (1997). The chemical modification of K-Ca channels by carbon monoxide in vascular smooth muscle cells. J. Biol. Chem..

[24] Hou S., Xu R., Heinemann S.H., Hoshi T. (2008). The RCK1 high-affinity Ca2 + sensor confers carbon monoxide sensitivity to Slo1 BK channels. Proc. Natl. Acad. Sci. U. S. A..

[25] Armstrong F.A. (2004). Hydrogenases: active site puzzles and progress. Curr. Opin. Chem. Biol..

[26] Stripp S.T., Goldet G., Brandmayr C., Sanganas O., Vincent K.A., Haumann M., Armstrong F.A., Happe T. (2009). How oxygen attacks [FeFe] hydrogenases from photosynthetic organisms. Proc. Natl. Acad. Sci. U. S. A..

[27] Kuiper H.A., Lerch K., Brunori M., Finazzi Agro A. (1980). Luminescence of the copper-carbon monoxide complex of *Neurospora* tyrosinase. FEBS Lett..

[28] Finazzi-Agro A., Zolla L., Flamigni L., Kuiper H.A., Brunori M. (1982). Spectroscopy of (carbon monoxy)hemocyanins. Phosphorescence of the binuclear carbonylated copper centers. Biochemistry.

[29] van der Deen H., Hoving H. (1979). An infrared study of carbon monoxide complexes of hemocyanins. Evidence for the structure of the co-binding site from vibrational analysis. Biophys. Chem..

[30] Taille C., El-Benna J., Lanone S., Boczkowski J., Motterlini R. (2005). Mitochondrial respiratory chain and NAD(P)H oxidase are targets for the antiproliferative effect of carbon monoxide in human airway smooth muscle. J. Biol. Chem..

[31] Tavares A.F.N., Teixeira M., Romao C.C., Seixas J.D., Nobre L.S., Saraiva L.M. (2011). Reactive oxygen species mediate bactericidal killing elicited by carbon monoxide-releasing molecules. J. Biol. Chem..

[32] D'mello R., Hill S., Poole R.K. (1996). The cytochrome *bd* quinol oxidase in *Escherichia coli* has an extremely high oxygen affinity and two oxygen-binding haems: implications for regulation of activity in vivo by oxygen inhibition. Microbiology.

[33] Calhoun M.W., Gennis R.B. (1993). Demonstration of separate genetic loci encoding distinct membrane-bound respiratory NADH dehydrogenases in Escherichia-coli. J. Bacteriol..

[34] Gunsalus R.P. (1992). Control of electron flow in Escherichia-Coli — coordinated transcription of respiratory pathway genes. J. Bacteriol..

[35] Tseng C.-P., Albrecht J., Gunsalus R.P. (1996). Effect of microaerophilic cell growth conditions on expression of the aerobic (*cyo*ABCDE and *cyd*AB) and anaerobic (*nar*GHJI, *frd*ABCD, and *dms*ABC) respiratory pathway genes in Escherichia coli. J. Bacteriol..

[36] Blattner F.R., Plunkett G., Bloch C.A., Perna N.T., Burland V., Riley M., ColladoVides J., Glasner J.D., Rode C.K., Mayhew G.F., Gregor J., Davis N.W., Kirkpatrick H.A., Goeden M.A., Rose D.J., Mau B., Shao Y. (1997). The complete genome sequence of *Escherichia coli* K-12. Science.

[37] Bekker M., de Vries S., Ter Beek A., Hellingwerf K.J., de Mattos M.J. (2009). Respiration of Escherichia coli can be fully uncoupled via the nonelectrogenic terminal cytochrome bd-II oxidase. J. Bacteriol..

[38] Poole R.K., Graham J.M., Higgins J.A. (1993). The isolation of membranes from bacteria.

[39] Markwell M.A.K., Haas S.M., Bieber L.L., Tolbert N.E. (1978). A modification of the Lowry procedure to simplify protein determination in membrane and lipoprotein samples. Anal. Biochem..

[40] Motterlini R., Clark J.E., Foresti R., Sarathchandra P., Mann B.E., Green C.J. (2002). Carbon monoxide-releasing molecules: characterization of biochemical and vascular activities. Circ. Res..

[41] Johnson T.R., Mann B.E., Teasdale I.P., Adams H., Foresti R., Green C.J., Motterlini R. (2007). Metal carbonyls as pharmaceuticals? [Ru(CO)_3_Cl(glycinate)], a CO-releasing molecule with an extensive aqueous solution chemistry. Dalton Trans..

[42] Alessio E., Mestroni G., Nardin G., Attia W.M., Calligaris M., Sava G., Zorzet S. (1988). Cis- and trans-dihalotetrakis(dimethyl sulfoxide)ruthenium(II) complexes (RuX2(DMSO)4; X = Cl, Br): synthesis, structure, and antitumor activity. Inorg. Chem..

[43] Lo Iacono L., Boczkowski J., Zini R., Salouage I., Berdaux A., Motterlini R., Morin D. (2011). A carbon monoxide-releasing molecule (CORM-3) uncouples mitochondrial respiration and modulates the production of reactive oxygen species. Free Radic. Biol. Med..

[44] McLean S., Mann B.E., Poole R.K. (2012). Sulfite species enhance carbon monoxide release from CO-releasing molecules: implications for the deoxymyoglobin assay of activity. Anal. Biochem..

[45] Santos-Silva T., Mukhopadhyay A., Seixas J.D., Bernardes G.J.L., Romao C.C., Romao M.J. (2011). CORM-3 reactivity toward proteins: the crystal structure of a Ru(II) dicarbonyl-lysozyme complex. J. Am. Chem. Soc..

[46] Desmard M., Foresti R., Morin D., Dagouassat M., Berdeaux A., Denamur E., Crook S.H., Mann B.E., Scapens D., Montravers P., Boczkowski J., Motterlini R. (2012). Differential antibacterial activity against Pseudomonas aeruginosa by carbon monoxide-releasing molecules. Antioxid. Redox Signal..

[47] McLean S., Begg R., Jesse H.E., Mann B.E., Sanguinetti G., Poole R.K. (2013). Analysis of the bacterial response to Ru(CO)3Cl(glycinate) (CORM-3) and the inactivated compound identifies the role played by the ruthenium compound and reveals sulfur-containing species as a major target of CORM-3 action. Antioxid. Redox Signal..

[48] Kalnenieks U., Galinina N., Bringer-Meyer S., Poole R.K. (1998). Membrane D-lactate oxidase in *Zymomonas mobilis*: evidence for a branched respiratory chain. FEMS Microbiol. Lett..

[49] Wood P.M. (1984). Bacterial proteins with CO-binding b- or c-type haem. Functions and absorption spectroscopy. Biochim. Biophys. Acta.

[50] Gilberthorpe N.J., Poole R.K. (2008). Nitric oxide homeostasis in *Salmonella typhimurium* — roles of respiratory nitrate reductase and flavohemoglobin. J. Biol. Chem..

[51] Pirt S.J. (1985). Principles of Microbe and Cell Cultivation.

[52] Shiva S., Huang Z., Grubina R., Sun J., Ringwood L.A., MacArthur P.H., Xu X., Murphy E., Darley-Usmar V.M., Gladwin M.T. (2007). Deoxymyoglobin is a nitrite reductase that generates nitric oxide and regulates mitochondrial respiration. Circ. Res..

[53] Hendgen-Cotta U.B., Merx M.W., Shiva S., Schmitz J., Becher S., Klare J.P., Steinhoff H.J., Goedecke A., Schrader J., Gladwin M.T., Kelm M., Rassaf T. (2008). Nitrite reductase activity of myoglobin regulates respiration and cellular viability in myocardial ischemia-reperfusion injury. Proc. Natl. Acad. Sci. U. S. A..

[54] D'mello R., Hill S., Poole R.K. (1994). Determination of the oxygen affinities of terminal oxidases in *Azotobacter vinelandii* using the deoxygenation of oxyleghaemoglobin and oxymyoglobin: Cytochrome *bd* is a low-affinity oxidase. Microbiology.

[55] Evans C.G.T., Herbert D., Tempest D.W., Norris J.R., Ribbons D.W. (1970). The continuous cultivation of microorganisms part 2 construction of a chemostat. Methods in Microbiology.

[56] Graham A.I., Hunt S., Stokes S.L., Bramall N., Bunch J., Cox A.G., McLeod C.W., Poole R.K. (2009). Severe zinc depletion of Escherichia coli: roles for high affinity zinc binding by ZinT, zinc transport and zinc-independent proteins. J. Biol. Chem..

[57] Mason M.G., Shepherd M., Nicholls P., Dobbin P.S., Dodsworth K.S., Poole R.K., Cooper C.E. (2009). Cytochrome bd confers nitric oxide resistance to Escherichia coli. Nat. Chem. Biol..

[58] Pudek M.R., Bragg P.D. (1974). Inhibition by cyanide of the respiratory chain oxidases of *Escherichia coli*. Arch. Biochem. Biophys..

[59] Poole R.K., Williams H.D., Downie J.A., Gibson F. (1989). Mutations affecting the cytochrome d-containing oxidase complex of Escherichia coli K12: identification and mapping of a fourth locus, cydD. J. Gen. Microbiol..

[60] Sturr M.G., Krulwich T.A., Hicks D.B. (1996). Purification of a cytochrome *bd* terminal oxidase encoded by the *Escherichia coli app* locus from a Dcyo Dcyd strain complemented by genes from *Bacillus firmus* OF4. J. Bacteriol..

[61] Borisov V.B. (2008). Interaction of bd-type quinol oxidase from Escherichia coli and carbon monoxide: heme d binds CO with high affinity. Biochemistry (Mosc).

[62] Cheesman M.R., Watmough N.J., Pires C.A., Turner R., Brittain T., Gennis R.B., Greenwood C., Thomson A.J. (1993). Cytochrome-bo from Escherichia-Coli — identification of haem ligands and reaction of the reduced enzyme with carbon monoxide. Biochem. J..

[63] Rice C.W., Hempfling W.P. (1978). Oxygen-limited continuous culture and respiratory energy conservation in *Escherichia coli*. J. Bacteriol..

[64] Kita K., Konishi K., Anraku Y. (1984). Terminal oxidases of Escherichia coli aerobic respiratory chain II. Purification and properties of cytochrome b558-d complex from cells grown with limited oxygen and evidence of branched electron-carrying systems. J. Biol. Chem..

[65] Kita K., Konishi K., Anraku Y. (1984). Terminal oxidases of Eschericha coli aerobic respiratory chain. I. Purification and properties of cytochrome b562-o complex from cells in the early exponential phase of aerobic growth. J. Biol. Chem..

[66] Bergersen F.J., Turner G.L. (1979). Systems utilizing oxygenated leghemoglobin and myoglobin as sources of free dissolved O_2_ at low concentrations for experiments with bacteria. Anal. Biochem..

[67] D'mello R., Hill S., Poole R.K. (1995). The oxygen affinity of cytochrome *bo′* in *Escherichia coli* determined by the deoxygenation of oxyleghemoglobin and oxymyoglobin: *K*_m_ values for oxygen are in the submicromolar range. J. Bacteriol..

[68] Nicholls D.G., Ferguson S.J. (2002). Bioenergetics 3.

[69] Smith H., Mann B.E., Motterlini R., Poole R.K. (2011). The carbon monoxide-releasing molecule, CORM-3 (Ru(CO)_3_Cl(Glycinate)), targets respiration and oxidases in *Campylobacter jejuni*, generating hydrogen peroxide. IUBMB Life.

[70] Poole R.K., Cook G.M. (2000). Redundancy of aerobic respiratory chains in bacteria? Routes, reasons and regulation. Adv. Microb. Physiol..

[71] Pullan S.T., Gidley M.D., Jones R.A., Barrett J., Stevanin T.A., Read R.C., Green J., Poole R.K. (2007). Nitric oxide in chemostat-cultured *Escherichia coli i*s sensed by Fnr and other global regulators: unaltered methionine biosynthesis indicates lack of S-nitrosation. J. Bacteriol..

[72] Borisov V.B., Forte E., Sarti P., Brunori M., Konstantinov A.A., Giuffre A. (2007). Redox control of fast ligand dissociation from Escherichia coli cytochrome bd. Biochem. Biophys. Res. Commun..

[73] Hill B.C., Hill J.J., Gennis R.B. (1994). The room temperature reaction of carbon monoxide and oxygen with the cytochrome bd quinol oxidase from Escherichia coli. Biochemistry.

[74] Smith A., Hill S., Anthony C. (1990). The purification, characterization and role of the d-type cytochrome oxidase of Klebsiella pneumoniae during nitrogen fixation. J. Gen. Microbiol..

[75] Jackson R.J., Elvers K.T., Lee L.J., Gidley M.D., Wainwright L.M., Lightfoot J., Park S.F., Poole R.K. (2007). Oxygen reactivity of both respiratory oxidases in *Campylobacter jejuni*: the *cydAB* genes encode a cyanide-resistant, low-affinity oxidase that is not of the cytochrome *bd* type. J. Bacteriol..

[76] Preisig O., Zufferey R., Thonymeyer L., Appleby C.A., Hennecke H. (1996). A high-affinity *cbb*_3_-type cytochrome oxidase terminates the symbiosis-specific respiratory chain of *Bradyrhizobium japonicum*. J. Bacteriol..

[77] Appleby C.A., Bergersen F.J., Bergersen F.J. (1980). Preparation and experimental use of leghaemoglobin. Methods for Evaluating Biological Nitrogen Fixation.

